# Biochar Decreases Cr Toxicity and Accumulation in Sunflower Grown in Cr(VI)-Polluted Soil

**DOI:** 10.3390/toxics11090787

**Published:** 2023-09-16

**Authors:** Shuai Li, Yiming Xie, Shuguang Jiang, Mingda Yang, Hongxia Lei, Wenzhi Cui, Fayuan Wang

**Affiliations:** 1 Institute of Resources, Environment, and Agricultural Product Quality and Safety, Shangqiu Academy of Agriculture and Forestry Sciences, Shangqiu 476000, China; hnsqlishuai@163.com (S.L.); xieyiming1506@163.com (Y.X.); 15236196390@139.com (S.J.); yangmingda1020@163.com (M.Y.); lhx1964@163.com (H.L.); 2 The Suihuang Laboratory, Shangqiu 476000, China; 3 College of Environment and Safety Engineering, Qingdao University of Science and Technology, Qingdao 266042, China; cwz2156411@163.com

**Keywords:** soil amendment, chromium pollution, soil immobilization, *Helianthus annuus* L., phytoremediation

## Abstract

Biochar is preferentially recommended for the remediation of heavy metal-polluted soils. Sunflower is an important high-biomass oil crop with a promising potential for phytoremediation of Cr(VI)-polluted soil. However, how biochar affects sunflower growth and Cr accumulation in Cr(VI)-polluted soil needs to be elucidated. Here, a pot culture experiment was conducted to study whether soil amendment with biochar (0, 0.1%, 1%, and 5%, *w*/*w*) can mitigate Cr toxicity and accumulation in sunflower seedlings grown in soils artificially polluted with different levels of Cr(VI) (0, 50, and 250 mg Cr(VI)/kg soil). The addition of Cr(VI) exhibited significant phytotoxicity, as evidenced by inhibited plant growth and even the death of seedlings at 250 mg/kg Cr(VI). Overall, biochar amendment showed positive effects on plant growth and Cr immobilization, dependent on both the biochar dose and Cr addition level. When 50 mg/kg Cr(VI) was added, 1% biochar showed positive effects similar to 5% biochar on improving plant growth and mineral nutrition (particularly K), reducing Cr content in shoots and roots, and decreasing Cr availability and Cr(VI) content in the soil. In comparison with non-amendment, 1% and 5% biochar caused 85% and 100% increase in shoot dry weights, and 75% and 86% reduction in shoot Cr concentrations, respectively. When 250 mg/kg Cr(VI) was added, a 5% dose produced much better benefits than 1%, while a 0.1% dose did not help plants to survive. Overall, an appropriate dose of biochar enhanced Cr(VI) immobilization and subsequently decreased its toxicity and accumulation in sunflower seedlings. Our findings confirm that biochar can be used as an efficient amendment for the remediation of Cr(VI)-polluted soils and cleaner production of sunflower oil and biomass.

## 1. Introduction

Chromium (Cr) is a common toxic metallic pollutant in soil. It has been estimated that more than 30,000 tons of Cr have been released into the environment globally in the past 50 years, most of which has accumulated in soils [1]. In a national survey of the soil contamination status of China, 1.1% of soil samples had Cr concentrations exceeding the environmental quality standard [1]. A meta-analysis shows that Cr concentrations in China’s farmland soil range from 1.48 to 820.24 mg/kg [1]. According to China’s soil quality standard (GB15618-2018), about 4.31% and 0.12% of the sampling sites exceed the screening values (150–250 mg/kg) and the intervention values (800–1300 mg/kg), respectively [1]. In the natural environment, Cr(VI) and Cr(III) are the most common and stable forms. Cr(III) is an essential trace element for human and animal health, but not required by the plants [2]. Differently, Cr(VI) has been classified as a class I carcinogen to humans [3]. Cr in the agricultural soils of China shows high non-cancer and cancer health risks [4]. In agricultural soil, excessive Cr(VI) can not only produce phytotoxicity, but also accumulate in edible crop tissues, consequently causing health risks via the food chain [5]. Considering the above context, effective technologies need to be exploited to remediate Cr(VI)-polluted soils. 

Biochar is a type of carbon-rich material with rich porous structures, multiple functional groups, and a high adsorption capacity. Previous studies have confirmed that biochar can be used as a soil amendment for the remediation of soils polluted with heavy metal(loid)s via various mechanisms, such as electrostatic adsorption, ion exchange, complexation, and precipitation [6,7,8]. Via the immobilization and reduction of Cr(VI), biochar generally decreases the availability and toxicity of Cr(VI) to plants and thus reduces Cr accumulation in plant tissues [9,10,11,12]. However, because plants differ in their Cr detoxification capacity and mechanisms, the toxicity and uptake of Cr in plants vary with the type of plant species [2]. In addition to pollution abatement, biochar can improve the soil’s physical, chemical, and biological properties and fertility, promoting plant performance in polluted soils [13,14,15,16,17]. A meta-analysis estimates that the overall effect of biochar on plant productivity can reach 16.0 ± 1.26%, but plant productivity response varies from −31.8% to 974%, largely dependent on biochar properties and soil conditions [15]. The effects of biochar can change under different soil conditions [18]. One study found that the application of 5% biochar decreased the bioavailability of Cu, Cd, Ni, and Zn more effectively in acidic soil than in alkaline soil [19]. A meta-analysis shows that biochar produced the greatest benefit for crop productivity in sandy soils, followed by clay, then silt or loam-textured soils [20]. 

Sunflower generally has a strong heavy metal enrichment capacity and thus is recommended for the phytoremediation of heavy metals, including Cr [21,22,23,24]. Meanwhile, sunflower is a bioenergy crop widely grown to produce bio-oils [24]. In a Cu-polluted soil, amendment with biochar significantly decreased leachable soil Cu and increased sunflower shoot biomass [25]. Another study showed that the application of tannery solid waste biochar loaded with microbe (*Trichoderma* and *Bacillus*) promoted sunflower growth [26]. Choppala et al. [12] found that chicken waste biochar (5%, *w*/*w*) was less effective than black carbon in reducing the phytotoxicity of Cr(VI) in sunflower; however, considering the authors used only one type and one dose, the effects of biochar cannot be concluded imprudently. How biochar affects Cr toxicity and its effects on sunflower performance needs to be explored. 

We hypothesized that the application of biochar can enhance the immobilization of Cr in soil and reduce its toxicity to sunflower, accordingly improving plant performance in Cr-polluted soil. Thereby, using oil sunflower as a test plant and different doses of biochar, we conducted a pot culture experiment to verify whether biochar can decrease Cr phytotoxicity and accumulation in sunflower. Our ultimate aim is to select an appropriate dose of biochar for the remediation of soils with different Cr-pollution levels. Our results can also help to recognize whether biochar facilitates the phytoremediation of Cr using sunflower and the safe production of sunflower in Cr-polluted soil.

## 2. Materials and Methods

### 2.1. Test Soil

The test soil was sampled from the surface layer (0–20 cm depth) of Xuezhuang experimental field of Shangqiu Academy of Agriculture and Forestry Sciences, located in Shuangba Town, Shangqiu, Henan Province, China. The soil was air-dried for two weeks and sieved through a 2 mm mesh for the plant culture experiment. The soil type was fluvo-aquic soil, with soil pH 8.46 (water soil ratio = 2.5:1), 1.11% organic matter, 1.36 g/kg total N, 1.02 g/kg total P, 11.49 g/kg total K, 58.28 mg/kg alkali-hydrolyzable N, 22.44 mg/kg available P, 136.00 mg/kg NH_4_OAc extractable K, 65.00 mg/kg total Cr, and 2.28 mg/kg ethylene diamine triacetic acid (EDTA)-extractable Cr.

### 2.2. Biochar

Commercial biochar was purchased from Lvzhiyuan Activated Carbon Co., Ltd. (Pingdingshan, China). The biochar was made by heating agricultural waste, such as straws of wheat and maize, woodchips, and fruit shells under a 500–600 °C limited-oxygen environment. The basic properties were as follows: pH 7.3, average particle size ≤ 75 μm, average specific surface area 863 m^2^/g, ash content 9%, moisture content 8.5%, methylene blue adsorption 60 mL/g, and iodine value 835 mg/g. The content of toxic metals (i.e., Pb, Cd, and Cr) in the biochar was below the limit of detection.

### 2.3. Experimental Design and Procedure

The pollution level of Cr was simulated based on the environmental Cr-pollution status [1] and China’s soil environmental quality standard (GB15618-2018). A bi-factorial experiment was designed, including three Cr addition levels (0, 50, and 250 mg Cr(VI)/kg soil) and four biochar doses (0, 0.1%, 1%, and 5%, *w*/*w*). Each treatment had four replicates. A proper amount of K_2_Cr_2_O_7_ powder was weighed and mixed thoroughly into the test soil (see Section 2.1) to achieve the target levels and then biochar was mixed into the soil at the designed doses. Thereafter, 1500 g soil with or without biochar was filled into each pot (top diameter 17 cm, bottom diameter 13 cm, and height 15 cm). The pots were irrigated with tap water to maintain soil moisture at the maximum water holding capacity and placed in a naturally controlled greenhouse to equilibrate for 30 days. 

Eight seeds of oil sunflower (*Helianthus annuus* L. var. Shengyou GW-S7070) surface-sterilized in 10% H_2_O_2_ solution for ten minutes were grown in each pot and five seedlings were kept one week after seedling emergence. The seedlings were placed in the above-mentioned greenhouse with day/night temperature 26 ± 10 °C/20 ± 8 °C. Deionized water was irrigated to maintain a soil moisture of about 70% water-holding capacity for plant requirements. Weeds were eradicated timely after their emergence. No fertilizers were supplied during the whole growth period. 

### 2.4. Determination of Metals and Nutrients in Soil and Plant Samples

Shoots and roots were harvested separately eight weeks after sowing. After being washed with tap water and deionized water respectively, plant samples were oven-dried at 70 °C to constant weight and then weighed using a digital electronic balance to record the dry weights. The ground plant samples were digested using HNO_3_ (98%, guaranteed reagent grade) in a graphite digester. Cr and K concentrations in the digested solution were determined with a flame atomic absorption spectrophotometer (FAAS, AA-7000, Shimadzu, Kyoto, Japan). P concentration was determined using molybdenum antimony spectrophotometry using an ultraviolet visible spectrophotometer (UV-5100, Shanghai Metash Instruments Co., Ltd., Shanghai, China). Plant N content was determined using a Kjeldahl nitrogen analyzer (Hanon K9840, Shandong Hanon Instruments Co., Ltd., Jinan, China) after digesting with H_2_SO_4_ and H_2_O_2_. For quality assurance of the determination of Cr, N, P, and K in plants, blanks and standard plant materials [GBW-07603 (GSV-2)] and an external certified standard were used. The soil from each pot was mixed evenly and 200 g of soil was sampled and air-dried for analysis of soil pH and Cr availability. Soil pH (soil–water ratio, 1:2.5, *w*/*v*) was determined using a pH meter. The available Cr concentration was determined using FAAS after extraction using an EDTA solution [27]. Soil Cr(VI) concentration was determined using the 1,5-diphenylcarbazide spectrophotometric method (GB/T 15555.4-1995). The quantification limit of FAAS was 0.005 mg/L.

### 2.5. Data Analysis

The total uptake of Cr (mg) by the plant tissues was calculated by the product of plant dry weights and element concentrations in the plant tissues. The bioconcentration factor (BCF) was obtained by calculating the ratio of Cr concentration in plant tissues and soil total Cr concentration. The translocation factor (TF) was calculated to measure the degree of translocation of Cr from the roots to aerial parts.

BCF = Cr concentration in shoots or roots/Total Cr concentration in soil (1)

TF = Cr concentration in shoots/Cr concentration in roots(2)

The data were submitted to Excel 2010 and SPSS 19.0 for analysis. The results were presented as mean ± standard deviation (SD). A one-way ANOVA followed by a Duncan’s multiple range test (*p* < 0.05) were used to compare the significance of difference. Two-way ANOVA was used to analyze the main effect and the interactions between the Cr addition level and biochar dose.

## 3. Results

### 3.1. Plant Growth

**Figure 1:**
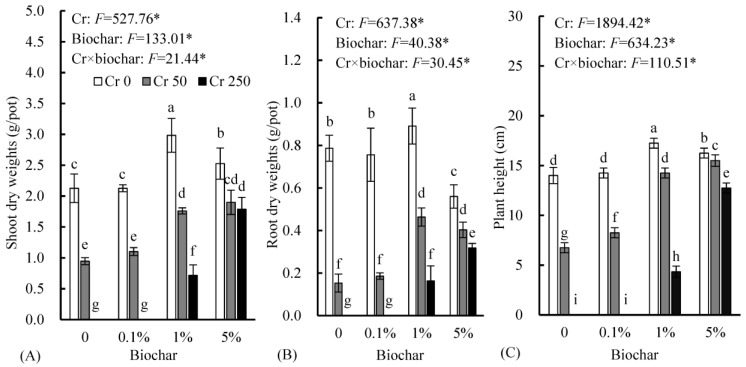
Effects of biochar on shoot (**A**) and root (**B**) dry weights and plant height (**C**) of oil sunflower seedlings in different Cr treatments. Cr0, Cr50, and Cr250 represent the treatments added with 0, 50, and 250 mg/kg Cr(VI), respectively. The numbers below *X*-axis (0, 0.1%, 1%, and 5%) represent biochar doses. Data are shown in mean ± SD. Different letters above the bars indicate significant differences among all the treatments using a one-way ANOVA followed by a Duncan’s multiple range test (*p* < 0.05). Significance level: * *p* < 0.01.

Irrespective of the biochar amendment, the addition of Cr significantly decreased the shoot and root dry weights of sunflower (Figure 1). The seedlings died soon after emergence at 250 mg/kg Cr addition level with 0 or 0.1% biochar (Figure S1). At 50 mg/kg Cr addition level, biochar did not significantly affect plant growth at 0.1%, but greatly improved both shoot and root dry weights and plant height at 1% and 5%. At 250 mg/kg Cr addition level, 5% biochar resulted in higher shoot and root dry weights and plant height than 1% biochar, whereas 0.1% biochar did not successfully help seedlings to survive. The two-way ANOVA results showed significant independent and interactive effects of Cr and biochar on both dry weights and height (Figure 1).

### 3.2. Plant Nutrients

Overall, Cr addition negatively affected shoot and root N, P, and K uptake (Table 1). The most pronounced decrease was observed in shoot and root K uptake. In most cases, biochar increased plant N, P, and K uptake, particularly at 1% and 5% doses. At 50 mg/kg Cr addition level, shoot and root K uptake in the plants with 1% and 5% biochar increased by 1.6–2.1 and 21.8–23.4 times, respectively, compared to non-amendment. At 250 mg/kg Cr addition level, 5% biochar resulted in higher shoot and root N and K uptake but lower root P uptake than 1% biochar. As for nutrient concentrations (%), when 50 mg/kg Cr was added, amendment with 1% and 5% biochar generally decreased root P concentration but increased root K concentration (Table S1). There were significant independent and interactive effects of Cr and biochar on N, P, and K concentrations and uptake in both shoots and roots.

### 3.3. Plant Cr Concentration and Uptake

Cr addition significantly increased Cr concentrations in plants, particularly in the roots (Table 2). In the treatments with 50 mg/kg Cr(VI) and no biochar, shoot and root Cr concentrations increased by 3.4 and 33 times, respectively, compared to the plants without Cr addition. At 50 mg/kg Cr addition level, in most cases, the biochar amendment caused remarkable reductions in shoot and root Cr concentrations, which gradually decreased with increasing biochar doses. Compared to non-amendment, 1% biochar caused 75% and 76% less Cr concentration in shoots and roots, respectively, and 5% biochar caused 86% and 81% reduction, respectively. 

Amendment with 1% and 5% biochar decreased shoot and root Cr uptake (Table 2). At 250 mg/kg Cr addition level, 5% biochar resulted in lower shoot and root Cr concentration and uptake than 1% biochar. Cr and biochar showed significant independent and interactive effects on both Cr concentration and uptake in both shoots and roots (Table 2). 

### 3.4. Bioconcentration and Translocation of Cr in Plants

Cr addition significantly increased the BCF, particularly in the roots (Table 3). In the treatments with 50 mg/kg Cr and no biochar, the BCF of Cr in shoots and roots increased by 1.5 and 24 times, respectively, compared to the plants without Cr addition. At 50 mg/kg Cr addition level, in most cases, the biochar amendment significantly decreased the BCF of Cr in shoots and roots, which gradually decreased with increasing biochar doses. At 50 and 250 mg/kg Cr addition levels, plants with 5% biochar had a lower BCF in shoots and roots than those with 1% biochar. Significant independent and interactive effects of Cr and biochar were observed on the BCF in both shoots and roots (Table 3).

Overall, the TF decreased with increasing Cr addition levels (Table 3). When no Cr was added, the F was decreased with increasing biochar doses. When 50 mg/kg Cr was added, biochar did not significantly change the TF. When 250 mg/kg Cr was added, the surviving plants had the lowest TF. 

### 3.5. Soil Available Cr and Cr(VI) Concentrations

Both soil available Cr and Cr(VI) concentrations were increased with increasing Cr addition levels (Table 4). Biochar amendment decreased the available Cr and Cr(VI) concentrations in the soil added with 250 mg/kg Cr, but did not show significant effects in the soil added with 0 and 50 mg/kg Cr. The two-way ANOVA results showed significant interactive effects on them between the Cr addition level and biochar dose (Table 4). 

### 3.6. Soil pH

**Figure 2:**
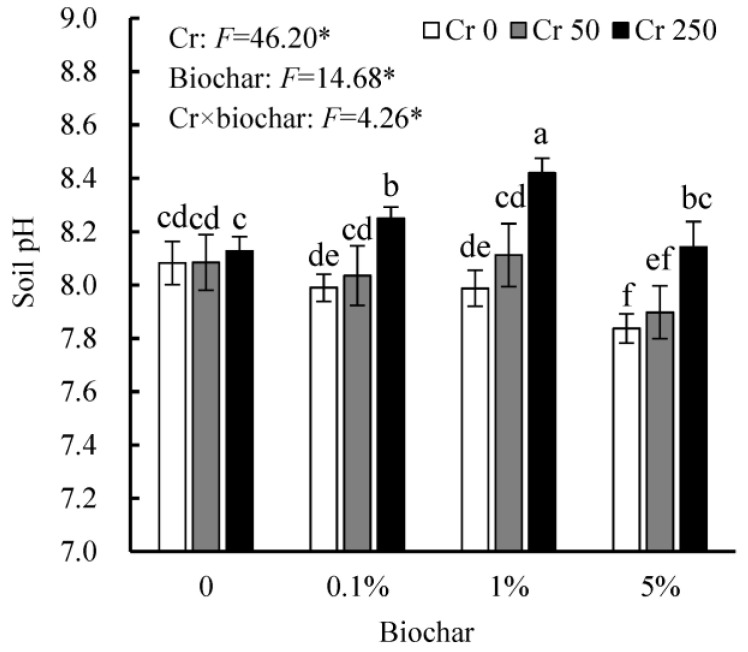
Effects of biochar on soil pH after harvesting oil sunflower seedlings in different Cr treatments. Cr0, Cr50, and Cr250 represent the treatments added with 0, 50, and 250 mg/kg Cr(VI), respectively. The numbers below *X*-axis (0, 0.1%, 1%, and 5%) represent biochar doses. Data are shown in mean ± SD. Different letters above the bars indicate significant differences among all the treatments using a one-way ANOVA followed by a Duncan’s multiple range test (*p* < 0.05). Significance level: * *p* < 0.01.

Cr addition did not affect the pH of the soil without the biochar amendment, but 250 mg/kg Cr(VI) increased soil pH when biochar was applied (Figure 2). In the soil added with 0 and 50 mg/kg Cr(VI), the biochar amendment had no significant effect on soil pH at 0.1% and 1% doses but decreased it at a 5% dose. When 250 mg/kg Cr(VI) was added, 0.1% and 1% biochar increased soil pH, and a 1% dose produced a more pronounced effect. 

## 4. Discussion

Cr is not an essential element required by plants. Excessive Cr, particularly Cr(VI), can cause a series of toxicity symptoms, including inhibited plant growth and development, biomass, induction of leaf chlorosis and necrosis, and physiochemical alterations (e.g., poor nutrition) [28]. Our present study confirmed the high toxicity of Cr(VI); 50 mg/kg Cr greatly inhibited plant growth and 250 mg/kg Cr caused seedlings to die. The first explanation can be ascribed to the uptake and accumulation of Cr in the oil sunflower. Generally, Cr is mainly accumulated in the roots which inhibits root growth and activity and further affects nutrient uptake and transportation to aerial parts, thereby inhibiting shoot growth [28]. Conspicuously, much lower root K concentrations were observed in some plants exposed to 50 and 250 mg/kg. Putatively, K deficiency may be another main cause of poor plant growth and seedling death induced by Cr. Cr(VI) can inhibit K^+^ uptake by maize root via interfering with the proton (H^+^) translocating mechanism [29]. Cr(III) can also inhibit the uptake of K^+^ in mash bean roots [30]. In addition, Cr has been widely confirmed to interfere with the uptake of various macronutrients (N, P, Ca, Mg, and S) and micronutrients (Fe, Mn, Cu, and Zn) [28,31]. We also found lower shoot P concentrations in the plants that received 50 mg/kg Cr. Although other nutrients were not determined, they are likely to be inhibited due to decreased root biomass by Cr. 

The most striking finding of our current study is that biochar effectively decreased Cr phytotoxicity in oil sunflower plants (Figure 1, Table 2). The plants that received biochar had better plant growth and survival in the soil polluted with Cr(VI). The benefits of biochar in soil–plant systems have been widely confirmed, including improved soil fertility and conditions, and better plant performance [15,32]. For heavy metal-polluted soil, biochar is evidenced to immobilize toxic metals including Cr via a range of mechanisms, such as adsorption, complexation, and precipitation, resulting in decreased bioavailability and uptake in plants [6,33]. Here, we observed that biochar decreased soil available Cr and Cr(VI) concentrations, and Cr accumulation in sunflower. Particularly, shoot Cr concentrations under 1% and 5% biochar amendment reached a normal range similar to non-polluted conditions (Table 2). These facts indicate that biochar can facilitate the immobilization and reduction of Cr(VI), which is in accordance with previous results [11,12,34]. Cr(VI) can be reduced to Cr(III) by the functional groups of biochar [35]. Zhong et al. [36] found that the –OH and –NH_3_ groups played a prominent role in electron donation, resulting in Cr(VI) reduction. Environmental persistent free radicals (EPFRs) on biochar can also participate in reducing Cr(VI) to Cr(III) [37]. In addition, sufficient nutrients are required for plants’ survival and growth under contamination conditions. The second reason for decreased phytotoxicity can be ascribed to better plant nutrition improved by biochar. One example is that biochar can help roots maintain a normal K concentration, which was severely disturbed by excessive Cr (Table S1). This is in accordance with many previous studies [38].

Another important finding is that the amelioration effects of biochar display a dose-dependent manner. Overall, 0.1% only showed some benefits for soil Cr immobilization, but not for plant growth, even under non-polluted conditions. In the soil with 50 mg/kg Cr(VI), 1% and 5% biochar produced similar benefits, whereas 5% biochar had better effects than a 1% dose at 250 mg/kg Cr addition level. Similar to our previous studies using soil polluted with Cd, Pb, and Zn [39,40], our current results evidence that 0.1% biochar is too low to remediate current Cr-polluted soils. In the soil with 50 mg/kg Cr(VI), 1% biochar was effective to reduce Cr phytotoxicity, but plant dry weights were not comparable to those under non-polluted conditions which implies a dose higher than 1% may be better. However, 5% biochar produced even lower root dry weights than 1% biochar, which indicates that 5% is too high for the remediation of 50 mg/kg Cr(VI). Sometimes, biochar may contain toxic substances, such as polycyclic aromatic hydrocarbons and heavy metals [41,42,43,44], which may negatively affect plants and soil biota. Differently, in the soil with severe Cr(VI) pollution (e.g., 250 mg/kg Cr(VI)), 5% biochar produced more positive effects on plant growth and soil Cr immobilization, suggesting that it is necessary to use a high dose of biochar to remediate severely Cr-polluted soil. In a word, in a Cr-remediation program using biochar, both pollution degree and biochar dosage should be clarified. 

Soil pH represents a key factor controlling Cr speciation, availability, and toxicity [2]. Because biochar is typically an alkaline material, the liming effect generally represents one of the most important mechanisms for the stabilization of heavy metals, especially in acidic soils [6,45]. Given that this study used an alkaline soil (pH 8.46) and biochar with a neutral pH (7.3), it is understandable that the biochar amendment did not increase but rather decreased the pH in the soil with 0 and 50 mg/kg Cr(VI). The liming effect is unlikely to be the main mechanism underlying biochar remediation of Cr(VI) in the present study. However, at 250 mg/kg Cr addition level, soil pH was significantly increased by the biochar amendment, particularly by 1% biochar. Soil pH is mediated by biochar and the redox of Cr. The reduction of Cr(VI) to Cr(III), which reacts strongly in the soil with 250 mg/kg Cr(VI), consumes H^+^ (Equation (3)), leading to a higher soil pH. Biochar amendment may increase soil pH via stimulating Cr(VI) reduction. Compared to the soil pH (8.46) before plant culture, Cr pollution and plant growth decreased soil pH, but was further mediated by biochar. The interactive effects of biochar and Cr(VI) can explain their complex impact on the pH of the soil with severe Cr(VI) pollution.

Cr_2_O_7_^2−^ + 14H^+^ + 6e^−^ →2Cr^3+^ + 7H_2_O (3)

Sunflower as a bioenergy crop has been highly recommended for the phytoremediation of heavy metal-polluted soils with additional advantages (biomass and oil) [46,47,48]. Our present results show that biochar amendment not only facilitated the stabilization of Cr, but also decreased BCF of Cr in shoots and roots of sunflower in Cr-polluted soil, indicating that biochar has a promising application in assisting the phytostabilization of Cr. Furthermore, only sunflowers with the biochar amendment survived in severely Cr(VI)-polluted soil, suggesting a potential of biochar for revegetation and ecological restoration. At 50 and 250 mg/kg Cr addition levels, shoot Cr concentrations after biochar amendment reached a normal range under non-Cr pollution. Due to its low TF, Cr accumulates mainly in sunflower roots but transports difficultly to aerial parts, resulting in a much lower concentration in the seeds than in the roots and shoots [49]. In summary, biochar can be considered an effective tool for the safe production of sunflower oil and biomass in Cr-polluted soils for human and animal consumption. In future, long-term field experiments are needed to explore the effects of biochar under varied environmental conditions. 

## 5. Conclusions

Overall, Cr(VI) pollution provoked a dose-dependent phytotoxicity in sunflower. Biochar amendment not only facilitated Cr(VI) immobilization and reduction, but also improved plant nutrition (particularly K), thus mitigating Cr accumulation and toxicity in plants. However, the effects of biochar were highly dependent on Cr(VI) pollution level and biochar dose. For the soil with 50 mg/kg Cr(VI), a 1% dose was as effective as a 5% dose for improving plant growth and nutrition, reducing tissue Cr content, and enhancing soil Cr(VI) immobilization, but as for the soil with 250 mg/kg Cr(VI), a 5% dose produced much better benefits. Particularly, the biochar amendment even decreased shoot Cr concentrations to a normal range under non-pollution conditions. Our results show that biochar can be employed as an effective amendment for the remediation of Cr(VI)-polluted soils and the safe production of sunflower soil and biomass, but the dose of biochar should be selected according to the pollution degree of Cr(VI).

## Figures and Tables

**Table 1:** Effects of biochar on N, P, and K uptake in oil sunflower seedlings in different Cr treatments.

Cr (mg/kg)	Biochar	N Uptake (mg)		P Uptake (mg)		K Uptake (mg)	
		Shoot	Root	Shoot	Root	Shoot	Root
0	0	35.74 (4.82) c	9.30 (1.06) c	16.77 (1.61) c	5.45 (0.79) b	67.88 (6.42) b	32.65 (2.37) a
	0.1%	35.26 (7.08) c	9.26 (2.32) c	15.45 (1.92) c	5.47 (1.21) b	62.73 (5.46) b	32.13 (4.91) a
	1%	47.89 (2.58) b	13.72 (1.82) a	24.65 (4.63) a	7.22 (1.13) a	117.33 (22.98) a	34.11 (2.55) a
	5%	64.28 (3.02) a	14.06 (1.48) a	22.08 (2.95) b	3.63 (0.65) c	108.55 (9.87) a	19.52 (2.07) b
50	0	22.58 (3.98) d	4.89 (1.28) d	5.01 (0.66) g	2.31 (0.72) d	24.82 (4.22) c	0.35 (0.17) d
	0.1%	28.80 (2.59) d	4.69 (0.53) d	7.35 (0.67) fg	2.59 (0.28) d	36.06 (3.30) c	0.39 (0.03) d
	1%	37.24 (5.78) c	12.15 (1.05) b	9.39 (1.16) ef	3.68 (0.63) c	64.63 (6.48) b	8.53 (1.01) c
	5%	49.88 (6.88) b	8.73 (0.77) c	12.62 (1.96) d	2.76 (0.37) cd	76.04 (8.30) b	7.97 (1.44) c
250	0	—	—	—	—	—	—
	0.1%	—	—	—	—	—	—
	1%	25.27 (0.27) d	5.54 (1.84) d	10.16 (0.05) de	3.65 (1.26) c	23.19 (6.27) c	0.66 (0.21) d
	5%	49.02 (5.79) b	8.31 (1.09) c	11.25 (0.96) de	2.23 (0.21) d	70.38 (9.36) b	7.54 (1.37) c
Two-way ANOVA	Cr	167.99 *	239.54 *	291.49 *	158.96 *	222.67 *	1853.19 *
	Biochar	177.56 *	119.08 *	74.12 *	33.70 *	109.36 *	16.59 *
	Cr × Biochar	9.86 *	7.51 *	6.29 *	10.99 *	7.73 *	71.08 *

— means no data because seedlings died in these treatments. Different letters in the same column mean significant differences among all the treatments at *p* < 0.05. Significance level: * *p* < 0.01.

**Table 2:** Effects of biochar on Cr concentrations and uptake in oil sunflower seedlings in different Cr treatments.

Cr (mg/kg)	Biochar	Cr Conc. (mg/kg)		Cr Uptake (mg/pot)	
		Shoot	Root	Shoot	Root
0	0	35.54 (1.78) c	24.50 (12.83) e	0.07 (0.00) d	0.01 (0.00) d
	0.1%	39.99 (3.04) c	25.44 (2.83) e	0.08 (0.01) d	0.02 (0.00) d
	1%	34.29 (2.03) c	28.19 (1.55) e	0.10 (0.00) c	0.03 (0.00) d
	5%	31.72 (2.41) c	41.80 (4.83) e	0.08 (0.01) d	0.02 (0.00) d
50	0	157.63 (49.83) b	832.16 (51.61) b	0.17 (0.05) a	0.13 (0.02) b
	0.1%	143.82 (8.72) b	556.83 (117.77) c	0.16 (0.02) a	0.13 (0.03) b
	1%	39.74 (4.19) c	198.66 (13.12) d	0.06 (0.01) de	0.08 (0.02) c
	5%	22.09 (0.84) c	161.99 (24.91) d	0.05 (0.01) e	0.06 (0.01) c
250	0	—	—	—	—
	0.1%	—	—	—	—
	1%	197.20 (18.48) a	2436.75 (206.66) a	0.13 (0.03) b	0.39 (0.14) a
	5%	24.66 (3.14) c	160.21 (32.71) d	0.04 (0.01) e	0.12 (0.01) b
Two-way ANOVA	Cr	81.96 *	895.38 *	99.55 *	79.55 *
	Biochar	54.35 *	831.75 *	16.86 *	59.87 *
	Cr × Biochar	154.16 *	1309.24 *	76.14 *	81.24 *

— means no data because seedlings died in these treatments. Different letters in the same column mean significant differences among all the treatments at *p* < 0.05. Significance level: * *p* < 0.01.

**Table 3:** Effects of biochar on BCF and TF of Cr in oil sunflower seedlings in different Cr treatments.

Cr (mg/kg)	Biochar	BCF		TF
		Shoot	Root	
0	0	0.55 (0.03) b	0.29 (0.11) fg	2.33 (0.21) a
	0.1%	0.61 (0.05) b	0.39 (0.04) f	1.60 (0.26) b
	1%	0.53 (0.03) b	0.43 (0.02) f	1.22 (0.07) c
	5%	0.49 (0.04) bc	0.64 (0.07) f	0.83 (0.09) d
50	0	1.37 (0.43) a	7.24 (0.45) b	0.16 (0.02) e
	0.1%	1.25 (0.08) a	4.84 (1.02) c	0.15 (0.01) e
	1%	0.35 (0.04) c	1.73 (0.11) d	0.19 (0.01) e
	5%	0.19 (0.01) d	1.41 (0.22) de	0.15 (0.00) e
250	0	—	—	—
	0.1%	—	—	—
	1%	0.63 (0.06) b	7.74 (0.66) a	0.08 (0.00) ef
	5%	0.08 (0.01) de	1.20 (0.25) e	0.06 (0.00) ef
Two-way ANOVA	Cr	140.33 *	834.24 *	1632.55 *
	Biochar	35.04 *	202.46 *	76.86 *
	Cr × Biochar	67.48 *	687.51 *	89.66 *

— means no data because seedlings died in these treatments. Different letters in the same column mean significant differences among all the treatments at *p* < 0.05. Significance level: * *p* < 0.01.

**Table 4:** Effects of biochar on soil available Cr and Cr(VI) concentrations in different Cr treatments.

Cr (mg/kg)	Biochar	Available Cr Conc. (mg/kg)	Cr(VI) Conc. (mg/kg)
0	0	0.61 (0.02) f	0.68 (0.08) f
	0.1%	0.28 (0.04) f	0.85 (0.13) f
	1%	0.49 (0.05) f	1.40 (0.22) f
	5%	0.75 (0.05) f	1.51 (0.21) f
50	0	5.71 (0.16) e	8.12 (0.73) e
	0.1%	4.56 (0.25) e	9.25 (0.23) e
	1%	4.27 (0.09) e	10.86 (0.81) e
	5%	4.49 (0.18) e	9.82 (0.75) e
250	0	103.64 (7.49) a	91.54 (2.84) a
	0.1%	96.44 (2.70) b	85.94 (9.28) b
	1%	31.22 (1.49) c	46.55 (1.97) c
	5%	27.58 (2.69) d	37.25 (2.91) d
Two-way ANOVA	Cr	6718.44 *	2112.20 *
	Biochar	740.49 *	98.28 *
	Cr × Biochar	720.72 *	113.93 *

Different letters in the same column mean significant differences among all the treatments at *p* < 0.05. Significance level: * *p* < 0.01.

## Data Availability

Not applicable.

## Supplementary Materials

The following supporting information can be downloaded at: https://www.mdpi.com/article/10.3390/toxics11090787/s1. Figure S1. The growth status of seedlings grown in the soil with 250 mg/kg Cr(VI). Table S1. Effects of biochar on N, P, and K concentrations in oil sunflower seedlings in different Cr treatments.

## References

[1] Li X., Zhang J., Ma J., Liu Q., Shi T., Gong Y., Yang S., Wu Y. (2020). Status of chromium accumulation in agricultural soils across China (1989–2016). Chemosphere.

[2] Shahid M., Shamshad S., Rafiq M., Khalid S., Bibi I., Niazi N.K., Dumat C., Rashid M.I. (2017). Chromium speciation, bioavailability, uptake, toxicity and detoxification in soil-plant system: A review. Chemosphere.

[3] Wang F., Yang W., Zheng F., Sun Y. (2018). Removal of Cr (VI) from simulated and leachate wastewaters by Bentonite-supported zero-valent iron nanoparticles. Int. J. Environ. Res. Public Health.

[4] Yang S., Zhao J., Chang S.X., Collins C., Xu J., Liu X. (2019). Status assessment and probabilistic health risk modeling of metals accumulation in agriculture soils across China: A synthesis. Environ. Int..

[5] Kapoor R.T., Mfarrej M.F.B., Alam P., Rinklebe J., Ahmad P. (2022). Accumulation of chromium in plants and its repercussion in animals and humans. Environ. Pollut..

[6] He L., Zhong H., Liu G., Dai Z., Brookes P.C., Xu J. (2019). Remediation of heavy metal contaminated soils by biochar: Mechanisms, potential risks and applications in China. Environ. Pollut..

[7] O’Connor D., Peng T., Zhang J., Tsang D.C., Alessi D.S., Shen Z., Bolan N.S., Hou D. (2018). Biochar application for the remediation of heavy metal polluted land: A review of in situ field trials. Sci. Total Environ..

[8] Lahori A.H., Guo Z., Zhang Z., Li R., Mahar A., Awasthi M.K., Shen F., Sial T.A., Kumbhar F., Wang P. (2017). Use of biochar as an amendment for remediation of heavy metal-contaminated soils: Prospects and challenges. Pedosphere.

[9] Dai L., Chen Y., Liu L., Sun P., Liu J., Wang B., Yang S. (2022). Effect of biochar on the uptake, translocation and phytotoxicity of chromium in a soil-barley pot system. Sci. Total Environ..

[10] Kumar A., Joseph S., Tsechansky L., Schreiter I.J., Schüth C., Taherysoosavi S., Mitchell D.R., Graber E.R. (2020). Mechanistic evaluation of biochar potential for plant growth promotion and alleviation of chromium-induced phytotoxicity in *Ficus elastica*. Chemosphere.

[11] Mandal S., Sarkar B., Bolan N., Ok Y.S., Naidu R. (2017). Enhancement of chromate reduction in soils by surface modified biochar. J. Environ. Manag..

[12] Choppala G., Bolan N., Megharaj M., Chen Z., Naidu R. (2012). The influence of biochar and black carbon on reduction and bioavailability of chromate in soils. J. Environ. Qual..

[13] Ding Y., Liu Y., Liu S., Li Z., Tan X., Huang X., Zeng G., Zhou L., Zheng B. (2016). Biochar to improve soil fertility. A review. Agron. Sustain. Dev..

[14] Murtaza G., Ahmed Z., Usman M., Tariq W., Ullah Z., Shareef M., Iqbal H., Waqas M., Tariq A., Wu Y. (2021). Biochar induced modifications in soil properties and its impacts on crop growth and production. J. Plant Nutr..

[15] Dai Y., Zheng H., Jiang Z., Xing B. (2020). Combined effects of biochar properties and soil conditions on plant growth: A meta-analysis. Sci. Total Environ..

[16] Cui W., Liu Y., Li W., Pei L., Xu S., Sun Y., Liu J., Wang F. (2023). Remediation agents drive bacterial community in a Cd-contaminated soil. Toxics.

[17] Doan T.T., Henry-des-Tureaux T., Rumpel C., Janeau J.-L., Jouquet P. (2015). Impact of compost, vermicompost and biochar on soil fertility, maize yield and soil erosion in Northern Vietnam: A three year mesocosm experiment. Sci. Total Environ..

[18] Chen D., Liu X., Bian R., Cheng K., Zhang X., Zheng J., Joseph S., Crowley D., Pan G., Li L. (2018). Effects of biochar on availability and plant uptake of heavy metals—A meta-analysis. J. Environ. Manag..

[19] Golia E.E., Aslanidis P.-S.C., Papadimou S.G., Kantzou O.-D., Chartodiplomenou M.-A., Lakiotis K., Androudi M., Tsiropoulos N.G. (2022). Assessment of remediation of soils, moderately contaminated by potentially toxic metals, using different forms of carbon (charcoal, biochar, activated carbon). Impacts on contamination, metals availability and soil indices. Sustain. Chem. Pharmacy.

[20] Liu X., Zhang A., Ji C., Joseph S., Bian R., Li L., Pan G., Paz-Ferreiro J. (2013). Biochar’s effect on crop productivity and the dependence on experimental conditions—A meta-analysis of literature data. Plant Soil.

[21] Chen L., Yang J.-y., Wang D. (2020). Phytoremediation of uranium and cadmium contaminated soils by sunflower (*Helianthus annuus* L.) enhanced with biodegradable chelating agents. J. Clean. Prod..

[22] Alaboudi K.A., Ahmed B., Brodie G. (2018). Phytoremediation of Pb and Cd contaminated soils by using sunflower (*Helianthus annuus*) plant. Ann. Agric. Sci..

[23] Sudha M., Kanmani S. (2009). Phytoremediation of chromium contaminated soils using *Helianthus annuus* (sunflower). J. Ecotoxicol. Environ. Monit..

[24] Nguyen D.T.C., Nguyen T.T., Le H.T., Nguyen T.T.T., Bach L.G., Nguyen T.D., Vo D.-V.N., Van Tran T. (2021). The sunflower plant family for bioenergy, environmental remediation, nanotechnology, medicine, food and agriculture: A review. Environ. Chem. Lett..

[25] Jones S., Bardos R.P., Kidd P.S., Mench M., de Leij F., Hutchings T., Cundy A., Joyce C., Soja G., Friesl-Hanl W. (2016). Biochar and compost amendments enhance copper immobilisation and support plant growth in contaminated soils. J. Environ. Manag..

[26] Younas H., Nazir A., Bareen F. (2022). Application of microbe-impregnated tannery solid waste biochar in soil enhances growth performance of sunflower. Environ. Sci. Pollut. Res..

[27] Zeng F., Ali S., Zhang H., Ouyang Y., Qiu B., Wu F., Zhang G. (2011). The influence of pH and organic matter content in paddy soil on heavy metal availability and their uptake by rice plants. Environ. Pollut..

[28] Singh H.P., Mahajan P., Kaur S., Batish D.R., Kohli R.K. (2013). Chromium toxicity and tolerance in plants. Environ. Chem. Lett..

[29] Zaccheo P., Cocucci M., Cocucci S. (1985). Effects of Cr on proton extrusion, potassium uptake and transmembrane electric potential in maize root segments. Plant Cell Environ..

[30] Ahmad M.S.A., Hussain M., Alvi A.K., Kausar A. (2013). Potassium and calcium uptake in mashbean under lead and chromium stress. J. Plant Nutr..

[31] Oliveira H. (2012). Chromium as an environmental pollutant: Insights on induced plant toxicity. J. Bot..

[32] Joseph S., Cowie A.L., Van Zwieten L., Bolan N., Budai A., Buss W., Cayuela M.L., Graber E.R., Ippolito J.A., Kuzyakov Y. (2021). How biochar works, and when it doesn’t: A review of mechanisms controlling soil and plant responses to biochar. GCB Bioenergy.

[33] Wang Y., Liu Y., Zhan W., Zheng K., Wang J., Zhang C., Chen R. (2020). Stabilization of heavy metal-contaminated soils by biochar: Challenges and recommendations. Sci. Total Environ..

[34] Sun Y., Zheng F., Wang W., Zhang S., Wang F. (2020). Remediation of Cr(VI)-contaminated soil by nano-zero-valent iron in combination with biochar or humic acid and the consequences for plant performance. Toxics.

[35] Liang L., Xi F., Tan W., Meng X., Hu B., Wang X. (2021). Review of organic and inorganic pollutants removal by biochar and biochar-based composites. Biochar.

[36] Zhong M., Li M., Tan B., Gao B., Qiu Y., Wei X., Hao H., Xia Z., Zhang Q. (2021). Investigations of Cr (VI) removal by millet bran biochar modified with inorganic compounds: Momentous role of additional lactate. Sci. Total Environ..

[37] Zhao N., Yin Z., Liu F., Zhang M., Lv Y., Hao Z., Pan G., Zhang J. (2018). Environmentally persistent free radicals mediated removal of Cr(VI) from highly saline water by corn straw biochars. Bioresour. Technol..

[38] Hossain M.Z., Bahar M.M., Sarkar B., Donne S.W., Ok Y.S., Palansooriya K.N., Kirkham M.B., Chowdhury S., Bolan N. (2020). Biochar and its importance on nutrient dynamics in soil and plant. Biochar.

[39] Wang F., Cheng P., Zhang S., Zhang S., Sun Y. (2022). Contribution of arbuscular mycorrhizal fungi and soil amendments to remediation of heavy metal-contaminated soil using sweet sorghum. Pedosphere.

[40] Wang F., Zhang S., Cheng P., Zhang S., Sun Y. (2020). Effects of soil amendments on heavy metal immobilization and accumulation by maize grown in a multiple-metal-contaminated soil and their potential for safe crop production. Toxics.

[41] Freddo A., Cai C., Reid B.J. (2012). Environmental contextualisation of potential toxic elements and polycyclic aromatic hydrocarbons in biochar. Environ. Pollut..

[42] Fabbri D., Rombolà A.G., Torri C., Spokas K.A. (2013). Determination of polycyclic aromatic hydrocarbons in biochar and biochar amended soil. J. Analyt. Appl. Pyrolysis.

[43] Kusmierz M., Oleszczuk P., Kraska P., Palys E., Andruszczak S. (2016). Persistence of polycyclic aromatic hydrocarbons (PAHs) in biochar-amended soil. Chemosphere.

[44] Odinga E.S., Gudda F.O., Waigi M.G., Wang J., Gao Y. (2021). Occurrence, formation and environmental fate of polycyclic aromatic hydrocarbons in biochars. Fundam. Res..

[45] Zhang X., Wang H., He L., Lu K., Sarmah A., Li J., Bolan N.S., Pei J., Huang H. (2013). Using biochar for remediation of soils contaminated with heavy metals and organic pollutants. Environ. Sci. Pollut. Res..

[46] Mahmood A., Awan M.I., Sadaf S., Mukhtar A., Wang X., Fiaz S., Khan S.A., Ali H., Muhammad F., Hayat Z. (2022). Bio-diesel production of sunflower through sulphur management in a semi-arid subtropical environment. Environ. Sci. Pollut. Res..

[47] Zehra A., Sahito Z.A., Tong W., Tang L., Hamid Y., Khan M.B., Ali Z., Naqvi B., Yang X. (2020). Assessment of sunflower germplasm for phytoremediation of lead-polluted soil and production of seed oil and seed meal for human and animal consumption. J. Environ. Sci..

[48] Zhou J., Chen L., Peng L., Luo S., Zeng Q. (2020). Phytoremediation of heavy metals under an oil crop rotation and treatment of biochar from contaminated biomass for safe use. Chemosphere.

[49] Fozia A., Muhammad A.Z., Muhammad A., Zafar M.K. (2008). Effect of chromium on growth attributes in sunflower (*Helianthus annuus* L.). J. Environ. Sci..

