# Supplementary material for: Biochar Decreases Cr Toxicity and Accumulation in Sunflower Grown in Cr(VI)-Polluted Soil
Source: Toxics. 2023 Sep 16;11(9):787. doi: 10.3390/toxics11090787 (PMC10536207; doi:10.3390/toxics11090787)
Supplement: Supplementary file 1 [file toxics-11-00787-s001.zip › toxics-2582673-supplementary.pdf]

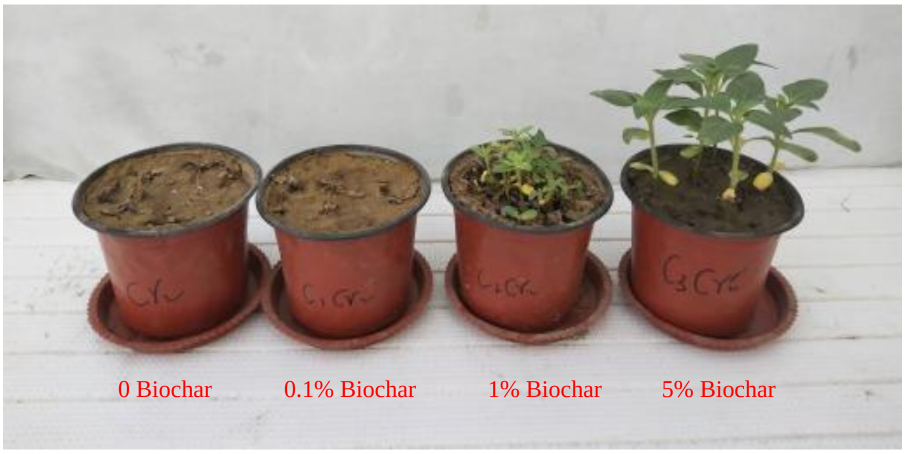

Figure S1. The growth status of seedlings grown in the soil with 250 mg/kg Cr(VI).

Table S1. Effects of biochar on N, P, and K concentrations in oil sunflower seedlings in different Cr treatments.

Cr (mg/kg)	Biochar	N conc. (%)		P conc. (%)		K conc. (%)	
		Shoot	Root	Shoot	Root	Shoot	Root
0	0	1.68(0.08)d	1.19(0.08)c	0.82(0.06)b	0.66(0.05)d	3.44(0.22)c	3.98(0.33)a
	0.1%	1.92(0.07)cd	1.22(0.12)c	0.82(0.05)b	0.72(0.08)d	3.34(0.20)c	3.56(0.48)bc
	1%	2.13(0.53)c	1.25(0.14)c	0.82(0.09)b	0.75(0.04)d	3.91(0.47)b	3.64(0.32)b
	5%	2.56(0.18)b	2.31(0.31)b	0.83(0.05)b	0.63(0.04)d	4.31(0.27)a	3.30(0.37)c
50	0	2.59(0.06)b	2.22(0.14)b	0.70(0.12)c	1.79(0.22)b	2.85(0.15)d	0.36(0.02)f
	0.1%	2.62(0.24)b	2.53(0.27)b	0.67(0.08)cd	1.51(0.15)c	3.28(0.26)c	0.22(0.01)fg
	1%	2.22(0.13)c	2.46(0.29)b	0.56(0.02)d	0.74(0.04)d	3.87(0.10)b	1.61(0.21)e
	5%	2.62(0.18)b	2.32(0.30)b	0.66(0.04)cd	0.66(0.04)d	4.00(0.14)ab	1.62(0.10)e
250	0	—	—	—	—	—	—
	0.1%	—	—	—	—	—	—
	1%	3.68(0.59)a	3.47(0.37)a	1.46(0.34)a	2.28(0.21)a	3.22(0.11)c	0.41(0.05)f
	5%	2.74(0.09)b	2.26(0.22)b	0.59(0.05)cd	0.70(0.08)d	3.93(0.11)b	2.48(0.29)d
Two-way ANOVA	Cr	60.23*	119.60*	58.32*	195.31*	415.47*	840.58*
	Biochar	104.43*	142.22*	79.28*	146.25*	279.21*	69.95*
	Cr × Biochar	85.93*	104.04*	102.31*	436.27*	81.03*	49.14*

— means no data, because seedlings died in these treatments. Different letters in the same column mean significant differences among all the treatments at $p < 0.05$. Significance level: *$p < 0.01$.
